# The roles of community nutrition scholars in changing mothers' child feeding, food preparation, and hygiene practices in southern Bangladesh

**DOI:** 10.3389/fpubh.2023.1135214

**Published:** 2023-06-15

**Authors:** Nozomi Kawarazuka, Farhana Ibrahim, Ebna Habib Md. Shofiur Rahaman, Gordon Prain

**Affiliations:** ^1^International Potato Center, The Consultative Group on International Agricultural Research (CGIAR), Hanoi, Vietnam; ^2^International Potato Center, The Consultative Group on International Agricultural Research (CGIAR), Dhaka, Bangladesh; ^3^Independent Consultant, The Consultative Group on International Agricultural Research (CGIAR), Cambridge, United Kingdom

**Keywords:** gender, micronutrient deficiencies, sweet potato, food-based approaches, Asian mega-deltas

## Abstract

**Introduction:**

This qualitative study evaluates a nutrition and hygiene education program led by trained community nutrition scholars for 5,000 mothers of small children in the Khulna and Satkhira districts in southern Bangladesh. The objectives of this study are as follows: (1) understanding the processes and reasonings behind mothers' improvement in child feeding, food preparation, hygiene, and homestead garden production, (2) understanding men's roles in facilitating women's behavioral changes, and (3) assessing the degree of changes in subjective notions of self-confidence, decision-making, and recognition among mothers and nutrition scholars.

**Methods:**

Data were collected through 14 focus group discussions with 80 participants and in-depth interviews with 6 women community nutrition scholars. Data was then analysed qualitatively by drawing on direct quotes from focus group discussions and interviews with detailed interpretation and account for respondents' behaviors and perceptions.

**Results:**

Overall findings confirm behavior changes by women, their spouses, and other family members. Many women were able to independently decide to change food allocation and child feeding practices after gaining self-confidence through the training. Men performed vital roles, such as purchasing nutritious food in local markets, providing labor for land preparation of homestead gardens, and defending the women from the resistance to change by their mothers-in-law.

**Discussion:**

While the study supports the literature that women's bargaining power in food/resource allocation is critical in child health and nutrition, the evaluation found that this process involves negotiations among family members. Engaging men and mothers-in-law in nutrition interventions have great potential to make nutrition interventions more effective.

## 1. Introduction

This study seeks to demonstrate the nuanced processes of changes involving gender relations in child feeding, food preparation, hygiene, and food production in homestead gardens. It is based on the evaluation of a 2.5-year gender-responsive intervention to support and improve the nutrition of small children through increasing the utilization of a beta-carotene-rich sweet potato variety, called orange-fleshed sweet potato (OFSP), and other nutrient-dense foods and through providing nutrition and health education (1). The nutrition intervention is part of a larger project titled “*Strengthening food system resilience in Asia's mega deltas with salt-tolerant sweet potato and potato”* in Khulna and Satkhira, southern Bangladesh.

Micronutrient deficiencies are a long-standing health problem in Bangladesh, especially among women of reproductive age and children under 5 years (2, 3). While supplements and food fortification distribution help increase the micronutrient intakes of target populations, promoting dietary diversity through food-based approaches is increasingly considered a key component of a sustainable response to micronutrient deficiencies in the global south (4, 5). The underlying causes of deficiencies are complex, involving a combination of biological, socio-economic, and environmental factors (6–9).

In rural Bangladesh, patriarchal gender norms limit female autonomy, decision-making power, education, mobility, and economic opportunities, and these restrictions are deeply internalized through everyday practices (10). Several studies in Bangladesh suggest that the subordinated position of women likely negatively affects the health and nutritional outcomes of children (11–13). Although women are responsible for child health and nutrition, whether they can improve childcare and feeding practices is dependent on the degree of influence from spouses and other family members. Hence, nutrition research and interventions need to consider gender relationships as well as engaging men who are influential as household decision-makers and gatekeepers (14, 15).

However, in nutrition research, “gender” is primarily focused on women, exploring the link between the degree of decision-making power and the outcomes of child nutrition. A recent systematic literature review on gender and child nutrition suggests that male engagement in child nutrition is missing as an indicator of female empowerment (16).

Similarly, gender is often equated with female participation in nutrition interventions. For example, homestead garden interventions have been promoted as sustainable alternatives to distributing micronutrient supplements in Bangladesh (17–19). Women are targeted for this initiative as they have relative autonomy in managing homestead gardens (20–22). Other studies suggest that nutrition education, provided to women with young children, can result in behavior changes in feeding practices, leading to improved food intake among young children (23–25).

Engaging directly with women and influencing decision-making regarding food choices and child feeding are theoretically aligned with the pathways for improving child nutritional outcomes (26); however, in practice, the process in which knowledge is translated into behaviors is not straightforward and is context specific. Negotiations with other household members must occur for women to change the allocation and composition of resources. Trade-offs may be involved in managing additional labor and time to improve the quality of childcare and diets.

Neglecting the process of intra-household negotiation risks placing all responsibilities on the woman and also risks reinforcing the overburden of childcare and domestic work on women. Recent literature from sub-Saharan Africa suggests that men's nutritional knowledge influences their children's nutritional and health outcomes (27). Men are also increasingly participating in activities that were previously considered under the domain of women (28). Furthermore, grandmothers (mothers-in-law) play significant roles in child feeding and childcare as care providers, advisers, and even supervisors (29, 30).

Therefore, it is essential for nutrition scholarship to consider broad gender relationships and communications within the household, particularly the roles and influences of other family members in childcare; providing training to women alone may not lead to behavioral changes if men and/or other family members are participating in household decision-making regarding child nutrition and health.

This nutrition intervention was conducted by trained women community nutrition scholars. The project employed local women as community nutrition scholars instead of hiring outside experts for two reasons. First, training by a female peer from the same or neighboring village provides a comfortable learning environment for young mothers. These community nutrition scholars can regularly visit trainee houses and provide face-to-face advice specific to the household situation. Second, the presence of female community nutrition scholars challenges stereotypical gender norms by establishing new practices, such as women taking a leadership role, speaking in public, and circulating the community without the accompaniment of husbands or male relatives. With these practices, this project sought to contribute to gender transformation.

The nutrition intervention consisted of three components: (1) increasing knowledge and skills regarding nutrition, food preparation, and child complementary feeding; (2) strengthening hygiene practices in the household and community; and (3) providing vegetable seeds and sweet potato planting materials together with homestead garden skills, such as the bed-planting technique. OFSPs contain carotenoids and β-carotene and have a great potential to increase the Vitamin A intake of young children, as well as adults, in a sustainable way (31, 32). Sweet potatoes can be easily and economically grown in homestead gardens where women have relative autonomy and control.

Husbands or male relatives of women participants were invited to the first training session during which community nutrition scholars provided an overview of the different training sessions and the benefits of women's participation. Approximately 50% of spouses/male relatives attended the first session. A total of 50 community nutrition scholars were trained during the project period between 2018–2020, and 5,000 women participated in this nutrition intervention.

The intervention evaluation had several research questions which this article addresses in the following three research questions:

1) To what extent have women improved everyday practices related to child feeding, food choices, food preparation, and homestead garden production, and why?
2) What are men's roles in facilitating women's behavioral change?
3) How much has the project contributed to increasing women's decision-making power, self-confidence, and recognition by their households and the community?

Subjective notions of decision-making power, self-confidence, and increased recognition by family members of the mothers who participated were assessed qualitatively through FGDs; these were supplemented with the observations of men from households where women had participated in the training as well as the observations of community nutrition scholars. The assessments of the community nutrition scholars were based on their experiences captured through FGDs and in-depth interviews.

The structure of this article is as follows: the next section outlines the research context and methods; the results section describes findings from the assessment in relation to the aforementioned three key research questions; and the discussion section draws on relevant literature pertaining to gender, agriculture, and nutrition and highlights the importance of considering gender in agricultural and nutrition interventions. We conclude by acknowledging and identifying knowledge gaps and further research required in gender and nutrition scholarship in the global south.

## 2. Research context and methods

### 2.1. Research context

**Figure 1:**
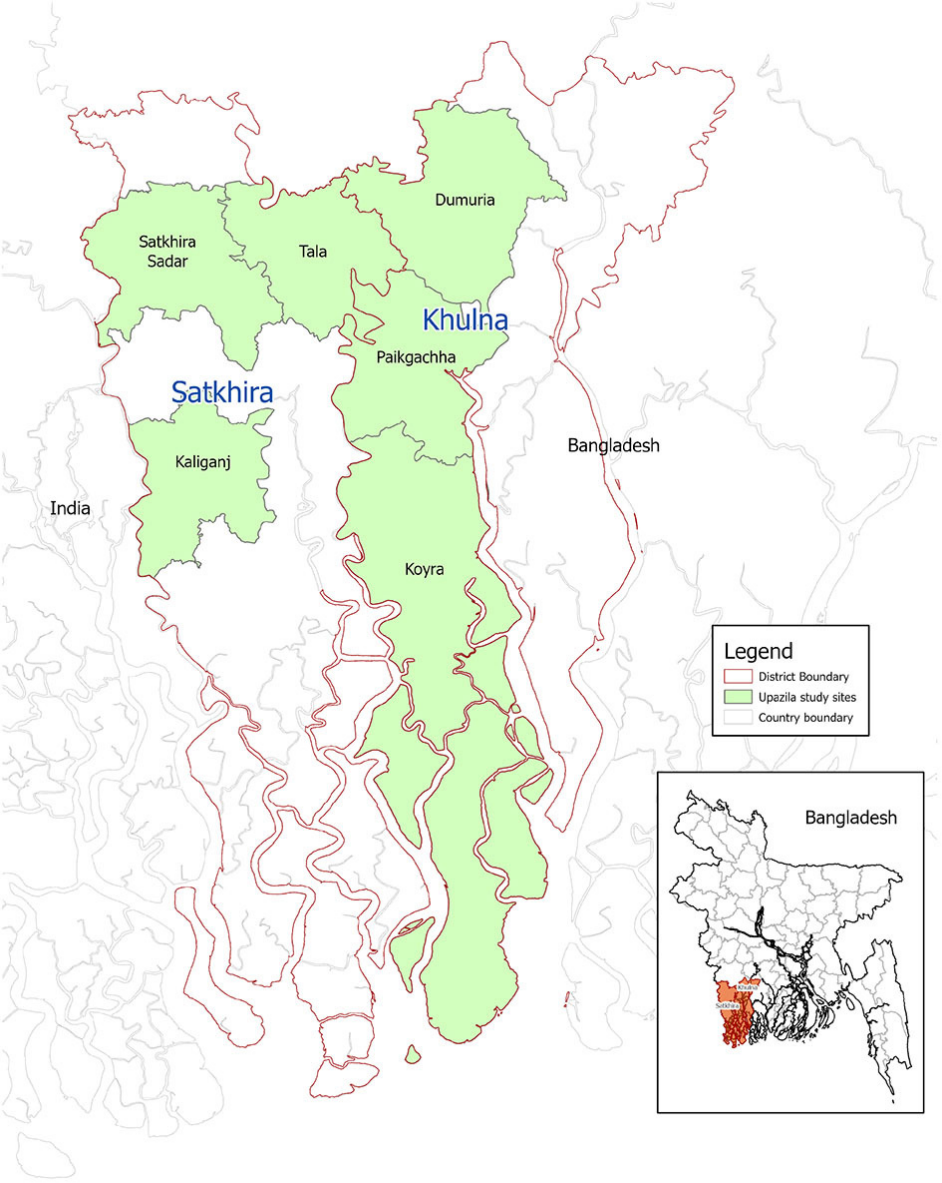
Map of Khulna and Satkhira district.

The study sites are the Khulna and Satkhira districts of the Ganges Brahmaputra Meghna delta region of Bangladesh (Figure 1) where children are highly vulnerable to undernutrition and micronutrient deficiencies as deltaic livelihoods are affected by environmental and climate changes (33), including frequent exposure to floods that cause outbreaks of infectious diseases (34–36). Compared to major cities in Bangladesh, the population density of both districts is low. However, urbanization is occurring in Khulna (34%) and Satkhira (10%). Islam is a major religion followed in both districts, but 23% are Hindus in Khulna (37).

In this region, soil salinity increases every year between November and May (Rabi/winter and Kharif I seasons). Agriculture in the dry winter season is particularly difficult due to the pressures caused by drought compounded with the salinity increase. Farmers often leave farms fallow until the next monsoon rainfall, which washes away salts accumulated in the farms (38).

Agriculture in this region is dominated by rice and fish/shrimp. Farmers often grow vegetables year-round in a dyke within the rice-fish cultivation system (39). Aside from rice, other crops, such as wheat, jute, grass pea, mustard, sunflower, watermelon, mung bean, sesame, potato, sweet potato, and different vegetables, are cultivated sporadically (40). Many farmers also raise livestock, such as cows, goats, sheep, poultry, and ducks, in their homesteads (41).

### 2.2. Research methods

The project evaluation was conducted in October 2020 through FGDs comprising three groups: (1) participants in the nutrition program, namely mothers with young children under 5 years; (2) the husbands or male family members of the women participants; and (3) the women community nutrition scholars. All participants come from different households. A total of 14 FGDs with 80 people (30 women, 30 men, and 20 women community nutrition scholars) were conducted. The details are described in Table 1.

**Table 1:** Focus group discussion demographics by location.

**District**	**Khulna**			**Satkhira**			**Total**
**Sub-district**	**Koyra**	**Dumuria**	**Paikgacha**	**Kaliganj**	**Satkhira Sadar**	**Tala**	**FGDs**
Women	1 (5)	1 (5)	1 (5)	1 (5)	1 (5)	1 (5)	6 (30)
Men	1 (5)	1 (5)	1 (5)	1 (5)	1 (5)	1 (5)	6 (30)
Community nutrition scholars	1 (10)			1 (10)			2 (20)

Numbers in parentheses represent the number of participants.

In addition, in-depth interviews were conducted with six community nutrition scholars (three from each district) in March 2021 by employing a most significant change approach, which is a qualitative method used to gain a deeper understanding of change through a project intervention (42). The participants of in-depth interviews are different from those who participated in the FGDs.

The fieldwork overlapped with the COVID-19 pandemic. While it was possible to travel from Dhaka to the study sites for face-to-face meetings with respondents, the sizes of FGDs were reduced to 5 people per discussion, and all discussions occurred outside or in a large open room. This created challenges in the quality of communication, particularly with participants wearing face masks and noise from passing cars and people. In two FGDs, villagers who were traveling unexpectedly participated in a discussion. Facilitators managed those challenges.

Compared to men participants, who were relaxed during the discussions, some women were initially nervous about publicly voicing their opinions. A female facilitator and note-taker worked to create a comfortable environment. Some women also participated in FGDs with their children. The mothers were occasionally distracted by their children during the discussion. Other women wanted to leave early to fulfill domestic responsibilities, such as preparing lunch for the children.

For community nutrition scholars, one FGD was organized in each district. The Khulna FGD was held in an empty, dark classroom. Community nutrition scholars arrived wet because there was heavy rain before the discussion. In Satkhira, participants gathered on the secluded porch of an empty house where there was sufficient light and privacy. Additional six in-depth interviews with community nutrition scholars were conducted in their houses.

There was a cultural challenge to conduct a fair evaluation and assessment by Bangladeshi participants. In the context of Bangladesh, people usually welcomed the project team as important guests and showed them great respect in part by avoiding making any criticisms. To make this project evaluation fair, including negative feedback, the facilitators of FGDs emphasized that the study was keen on learning from both positive and negative experiences, which would help improve future projects. The FGDs also included anonymous voting components which allowed participants to express opinions freely.

Our data analysis is primarily qualitative, drawing on direct quotes from focus group discussions through ethnographic analysis, a method that allows researchers to have detailed interpretation and account for respondents' behaviors and perceptions (43). First, the focus group questions and answers are sorted by key themes of our focus, including (1) the processes and reasonings behind mothers' improved or unimproved child feeding, food preparation, hygiene practices, and homestead garden production, (2) men's perceptions and roles in nutrition and child feeding, and (3) women's subjective notions of improvement in terms of self-confidence, decision-making, and recognition by their family and community. Second, within each theme, participants' answers were grouped into several common categories. In this process, similar answers were counted quantitatively to prove them as the majority responses. Third, commonalities and differences among the study sites were analyzed. In addition, notes from focus group discussions included non-verbal content, such as participants' attitudes and power dynamics, among participants. These were carefully reviewed to understand the background of which conversations were held.

In-depth interviews were analyzed based on themes such as changes in family relations, improved knowledge and skills, and intangible elements such as recognition and self-confidence. Quotations from focus group discussions and in-depth interviews were then revisited and reanalyzed by linking them to relevant literature.

## 3. Results

### 3.1. Changes in child feeding, food choices, food preparation, and homestead gardens

Overall findings suggest that the nutrition program was successful in improving mothers' practices related to child-feeding and healthcare, especially exclusively breastfeeding and appropriate complementary feeding practices in the first 1,000 days after the birth of a child. Women also changed vegetable preparation and cooking methods to minimize the loss of vitamins and minerals. All women who participated in FGDs planted vegetable seeds distributed during the training course and tried the bed-planting technique learned through training. Findings also confirm that many women have the decision-making power to change practices and control vegetables produced in homestead gardens.

Some participants mentioned mindset changes learned from the training, which facilitated behavioral changes:

“Now I think differently regarding nutrition. Not only children but mothers should also take nutritious food, such as calcium, vitamin, carbohydrate, etc. If you consume nutritious food, then you will be infected with less[sic] diseases.” (a woman FGD, Koyra, 15 October 2020).

“I think differently now. All of us became careful as to how to feed, what to feed the child to keep him healthy so that I and the family stay healthy.” (a woman FGD, Paikgacha, 19 October 2020).

Other important changes frequently mentioned by women were breastfeeding and complementary feeding practices for infants above 6 months. Changes include stopping exclusive breastfeeding after 6 months and preparing special baby food instead of providing the same food as adults. Several women mentioned that they used to exclusively feed rice.

“Earlier I did not care much about my child's food. I used to feed him whatever I cooked for the family, for example, rice, fish, and meat. Now, I cook separately for my child. I cook Khichadi mixed with all vegetables.” (a woman FGD, Koyra, 15 October 2020).

Many mothers mentioned being more attentive to child-feeding and spending more time preparing complementary food for weaning children:

“Before, my child did not want to eat. I did not use to give much time to feed her. Now I spend more time to feed her, and also I am conscious about timely feeding for each meal.” (a woman FGD, Kaliganj, 17 October 2020).

“I was not aware that children need additional food for their growth. Before I used to feed him less in terms of both quantity and frequency. Now I give him various food such as sweet potato vines, apples, noodles, and liver. I give him an egg every day. I did not feed my eldest son like this.” (a woman FGD, Satkhira Sadar, 18 October 2020).

Women were asked to identify the reasons they changed nutritional practices and the benefits gained from those changes. Out of the 30 women from six FGDs, 14 recognized health-related benefits as the main reason for continuing the new feeding methods. With great honesty, one woman mentioned the improvement of her son's health through the new feeding practices, which reduced whining. She claimed, “whenever my son is not healthy, he irritates.” She now recognizes that he is less irritable (a woman FGD, Kaliganj).

Seven mothers referred to weight and height gain as the major benefits, and two mothers identified improved memory and greater intelligence.

“(After changing complementary food), he is now growing up well. Both his weight and height are right according to the standard for his age. In a healthcare center, I was told that he is alright.” (a woman FGD, Kaliganj, 17 October 2020).

As mentioned by this woman, making special complementary food for their children after 6 months of age was an important change that was observed. Several women said that they used to give the same food cooked for their households to their children in smaller amounts. Additionally, many participants increased the number of feeding times from three to four per day. The most frequently mentioned special food is Khichadi, a rice and lentil dish with added vegetables. This basic dish is widely prepared, but the women describe a special preparation that is more nutritious.

“Earlier, I used to give my daughter only rice. Now I feed her milk and eggs. Also, I used to cook Khichadi with rice and pulses only. Now I add vegetables and eggs with fewer spices and less oil for her. Before I didn't have the knowledge, so I did not feed my eldest son in this way. I now give him seasonal fruits.” (a woman FGD, Kaliganj, 17 October 2020).

Out of the 30 FGD participants from six FGDs with women participants, 14 women made the same change as above when cooking Khichadi. In total, 13 women began to use eggs as complementary food. One woman mentioned that she used to sell all her duck and chicken eggs to local markets; now, she keeps some for her child.

Additional animal-source foods given to children as complementary food include milk, in both quantity and frequency, liver (mentioned in six FGDs), and a paste made from the heads of small fish and other fish products (mentioned in five FGDs). There was also increased consumption of leafy vegetables, including sweet potato vines as well as boiled sweet potato roots or potatoes (10 women).

“Now I ensure that every day we eat vegetables, including leafy vegetables such as red amaranth, aroid leaves, spinach, stem amaranth, kangkong, and sweet potato leaves. I did not know that these leafy vegetables are nutritious before.” (a woman FGD, Dumuria, 16 October 2020).

“Nobody in my household liked to eat vegetables before. I learnt[sic] that we should eat 250 grams of vegetables every day. Before we ate leafy vegetable[sic] if we wanted to. Since training[sic], I cultivate vegetables in the homestead garden no matter how hard it is and cook vegetables every day.” (a woman FGD, Tala, 20 October 2020).

Vegetables that women prepare for their households were primarily sourced from their homestead gardens. In three out of the six FGDs, all participants confirmed that they are self-sufficient in vegetable supply. In other cases, leafy vegetables came from the homestead garden, and other vegetables were purchased from a local market. Except in one case, all fruits were purchased from a local market.

Men FGDs confirmed that women shared information from the training with their husbands, and the most frequently shared knowledge was what types of food are nutritious, such as vegetables and fruits, and how to minimize the loss of nutrients during food preparation and cooking. Regarding the changes in wives' practices that men observed in the kitchen, food preparation ranked at the top, with detailed explanations including the methods to preserve nutrients, food safety, and hygiene practices.

“In my household, I observe that my wife changed cooking methods. Before training, she sliced vegetables finely and then washed them. Since training, she cleans them first with safe water and then cuts them into a bigger size. She also covers the pot during cooking, which prevents nutrients from being reduced by vapor. Earlier she did not do this.” (a man FGD, Satkhira Sadar, 18 October 2020).

“My wife used to keep food uncovered, which was unhygienic. She also cooked vegetables without considering the nutrient loss. But after training, she changed. She now always tries to give nutritious food, like different kinds of seasonal fruits, pulses, eggs, green peas, etc. She sometimes prepares mixed food with nutrient-rich vegetables.” (a man FGD, Dumuria, 16 October 2020).

All 30 women FGD participants planted vegetable seeds distributed by the community nutrition scholars. There were four common reasons why they planted the seeds: (1) to meet the nutritional needs of the household, especially vitamins and minerals; (2) to reduce food expenditure, especially during higher prices in local markets; (3) to consume safe, fresh, and pesticide-free vegetables; and (4) to earn extra income selling vegetables.

Women in the Dumuria FGD discussed giving vegetables as gifts to relatives or neighbors, which brought happiness to the giver and recipient and was another reason for producing vegetables in their homestead. These findings confirm that women have a certain decision-making capacity and control over vegetables produced in their homestead gardens.

In addition to vegetable seeds, women received sweet potato planting materials. Of the 30 participants, 29 confirmed that they now grow sweet potatoes in their homestead gardens. The woman who could not plant the vines said that she was not able to prepare the land in time, missing the season.

All participants from the 60 households consumed the vitamin A-rich OFSP produced in their homestead gardens and used them as complemental food for children. However, there is a concern about sustainability over time. To maintain these positive changes, a consistent supply of vegetable seeds and planting materials is required, for which the project is considering options, such as providing micro-credit.

While the findings highlight the positive aspects of nutrition interventions, four concerns that potentially weaken women's positions were identified through FGDs.

First, there was resistance by household members, especially mothers-in-law, to changing food preparation, food consumption patterns, and child feeding. Women shared experiences that their mothers-in-law did not understand the importance of changing practices. Several women said their husbands defended them and convinced their mothers by explaining the value of new knowledge and practices gained through the training.

When asked about their observation about the women's relationships with their mothers-in-law, community nutrition scholars shared some positive cases:

“The mothers-in-law encourage their daughters-in-law by saying ‘our time has already passed, and we should listen to new knowledge by training.' They think that the training is good for their daughters-in-law.” (Community Nutrition Scholar FGD, Satkhira, 21 October 2020).

Other women, for example, in Dumuria and Satkhira Sadar, mentioned the support they received from mothers- and fathers-in-law.

“My mother-in-law says, ‘I have grown somehow, your generation is growing in a better way now.' I also got books on childcare from my father-in-law.” (a woman FGD, a woman FGD, Dumuria, 16 October 2020).

Second, there was some resistance to the training course provided for women. In five FGDs, all men participants agree that there were negative reactions in the community, especially by older men, but perceptions eventually turned positive as more information became available.

“In this village, the general feelings of men about the training was good, people knew this was a good training. But very few people, those who are aged, had negative feelings as they always believe in the old traditions, and it was hard for them to accept new things. However, I think, day by day they became more informed about the training, and they no longer oppose this training.” (a man FGD, Paikgacha, 19 October 2020).

“Initially there was a negative idea (about the training) among other village members but when they came to know from me and other members who received training (in the first session) that the training topics were good and appropriate for the health and nutrition of family members they realized their mistake and were impressed and showed supportive attitudes to the organizer and others.” (a man FGD, Koyra, 15 October 2020).

The evidence above shows that men's participation in the first session of the training was very important to reassure other men in the community as well as in the household regarding mothers-in-law.

The third issue was the time constraints of women participants. Women needed to manage time for domestic tasks to attend training. Men reported that other household members, particularly mothers-in-law, took over domestic work and childcaring during the training. This could have caused tension in the household, although such negative responses were not reported during the FGDs. For future interventions, it may be helpful to invite mothers-in-law to the first session of the training and acknowledge their support for their daughters-in-law.

Finally, the training did not illicit positive outcomes for all participants equally. Community nutrition scholars highlighted that women from better-off households and those who were well-educated had deeper understandings of the new knowledge, greater improvements in their practices, and higher adoption rates of the new technologies. Nevertheless, overall adoption was high, which indicates that new knowledge and practices suggested in the training were adopted by less educated and poor women. Intra-household gender relations, such as lack of support from husbands and mothers-in-law, may have contributed to any lack of adoption. More detailed assessments of both household socio-economic data, as well as ethnographic research, are required to further understand underlying constraints.

Overall, the women trainees shared the knowledge from the training with their relatives and friends within and beyond their communities. There was good evidence from FGDs that information on child feeding, nutrition, and food preparation was widely disseminated through the women's social networks. Extrapolating from the dissemination efforts of the 5,000 women participants, a conservative scaling figure was estimated at 50,000 households.

### 3.2. Men's roles in facilitating behavioral changes of women

This sub-section illustrates men's changes in perceptions of child health and nutrition and their contributions to facilitating women's behavioral changes. Findings show that men played key factors in supporting women by providing labor for preparing land for homestead gardens, convincing their live-in mothers to support their wives in changing practices, and allocating home-produced cow milk and chicken eggs for home consumption instead of selling and/or purchasing nutrition-rich food from local markets.

During the FGDs with men, they were asked to describe two key points they learned in the first session of the nutrition training. In all FGDs, men reported (1) the importance of nutrition for pregnant and lactating women and (2) the specific food and care needed for small children, with one participant in Dumuria referring to the importance of the first 1,000 days.

Men confirmed that their attendance in the first session helped them to achieve positive images about the training, and, thereafter, they encouraged their wives to attend.

“I have learnt[sic] that the training provides a good thing that will be helpful for my family. They did not discuss bad things. This has increased my confidence (in the course) and that's why I encouraged my wife to participate in the training.” (a man FGD, Koyra, 15 October 2020).

A father-in-law who participated in the first session strongly supported his daughter-in-law to join the training:

“My daughter-in-law is very progressive about the training and very much interested in learning as she has a small child. I encouraged her to join the rest of the sessions.” (a man FGD, Dumuria, 16 October 2020).

Generally, men were positive about the women-targeted training despite not being invited to the remainder of the sessions. In three FGDs, men said that they were busy with agriculture, and it made sense to invite women. Other men highlighted that the topic of training was suitable for women as they are responsible for cooking and childcare. Some men in Dumuria suggested that it would be useful for men to attend some sessions so that they can better support their wives. A similar suggestion was made in Kaliganj that some shortened sessions for men would be helpful.

Men were asked about the contributions to household nutrition they made after the training. In the gender norms of the study sites, men usually go to local food markets. All six FGDs with male participants discussed that they buy nutritious fish, vegetables, and fruits in a market; one person specifically mentioned safe, pesticide-free vegetables. The importance of milk consumption was reviewed by men as well. Several men allocated more milk to their children instead of selling it, while others bought milk at a local market (11 references). Women's comments support the men's testimonies. All female participants confirmed that their spouses helped to buy nutritious food in a local market. A woman participant in the Paikgacha FGD stated, “we tell our husbands what to buy in a market. We tell him the benefits if he asks why.”

Another contribution to household nutrition security mentioned by men in all six FGDs was the production of nutrition-rich vegetables in homestead gardens. It was women who learned the bed-planting technique in the training, but men reported that their wives shared information on OFSP and vegetables, especially their high nutritional values as well as the bed-planting method. Men then helped their wives with specific activities, such as land preparation and planting. The following feedback by some men indicates men's perceived ownership and responsibility with vegetable production.

“After training, I cultivate...preparing bed and row and maintain row in my homestead gardening” (a man FGD, Koyra, 15 October 2020).

“I adopted the bed-planting technique as requested by my wife and planted seeds in the bed... The bed-planting technique made it easy for me to take care of vegetables and sweet potatoes” (a man FGD, Kaliganj, 17 October 2020).

“I helped my wife for[sic] land preparation. Both my wife and I worked together to plant sweet potato vines and vegetable seeds using the bed-planting technique by maintaining a distance between rows which I never did earlier. She regularly watered the homestead garden and added organic matter. No chemical fertilizers and pesticides were used. Therefore, ‘my' produce was completely safe that we consume them at home regularly.” (a man FGD, Paikgacha, 19 October 2020).

The above feedbacks were slightly conflicted with women's perceptions. As described in section 3.1, women said that they managed the homestead gardens, while many men show their ownership of the produce. Similarly, men also mentioned raising cows and poultry as their contribution to household nutrition, although, in the study sites, women raise livestock, including feeding and milking cows. This indicates that there is a perceptional gap between men and women regarding contributions to household nutrition and the notion of producing ownership; however, this is beyond the scope of this study. Understanding men's perceived contributions is important as it implies men's influences in these domains.

Men expressed an interest in the possibility of growing OFSP as a new commercial crop. However, initially, the vines were distributed to women to grow in homestead gardens for household consumption. In Satkhira Sadar, Tala, and Paikgach, men have some experience growing other sweet potato varieties on their farms, and they agree that OFSP roots could be a good commercial crop, while also cultivating them in homestead gardens for vegetable consumption. In Kaliganj, male participants said that there is a steady demand for sweet potatoes in their local markets. Some men, however, think that the growing season is too long for their crop rotation cycle. In Dumuria, men found that growing OFSP is easier and higher yielding compared to potatoes, although they were concerned that the price may be lower than that of potatoes. Thus, although the training was offered to women, men are certainly involved and interested in vegetable production activities, as they are responsible for agriculture.

In contrast, almost all men in the FGDs were not involved in OFSP food preparation and cooking as well as decisions associated with including OFSP in daily meals. A participant from Kaliganj mentioned:

“If a guest comes to my house that is the only time my wife discusses with me about food preparation. Otherwise, she does not. Most of the time we discuss about the food during eating, not before cooking. I have never discussed with my spouse about including OFSP in food preparation. Whenever she likes, she uses the leaves to make vegetables.” (a man FGD, Kaliganj, 17 October 2020).

Other participants agreed with his statement, and this confirms that food preparation and cooking remain in the women's domain.

The findings above confirm the importance of involving men in the training related to child health and nutrition as are key in food production for home consumption, food shopping, and decision-making. This should be a standard practice in future training and a major focus of effort. In the training, approximately 50% of spouses did not attend the first orientation session. Follow-up will be necessary to involve those men during other opportunities.

### 3.3. Improved women's positions in the household and the community

This assessment sought to explore how the knowledge and skills obtained through the training have empowered the women within the household as well as in the community. The female participants as well as the community nutrition scholars were asked to share their thoughts and feelings.

The results from the FGDs of women participants highlight strengthened self-confidence, which enabled them to improve childcare and food preparation practices. They now have a stronger voice in the family and have become more independent.

“In the past, I had to ask my husband for permission. Now we discuss together and consult each other. I used to be less careful, now when my child gets sick, I take him to the doctor right away. I have more awareness now.” (a woman FGD, Tala, 20 October 2020).

“My family has accepted me more. Now I can speak my mind in the family.” (a woman FGD, Dumuria, 16 October 2020).

“I planted the vegetable seeds I was given. I have managed to improve the earlier lack of vitamins. Now that I have got so much knowledge, I am able to move forward, I can make progress.” (a woman FGD, Paikgacha, 19 October 2020).

The community nutrition scholars reflected on the changes they observed among the female participants before and after the training in terms of confidence, autonomy, and decision-making. Scholars noticed their trainees became more confident by going out from home for training.

“Staying at home causes mothers to lose confidence. Attending the training made them step out of the door, which boosted their confidence. They are now producing vegetables on their homestead and supporting the family.” (an FGD with community nutrition scholars, Khulna, 22 October 2020).

“Mothers used to be shy; they covered their face and hesitated to speak in public. Now they talk freely and can go out on their own.” (an FGD with community nutrition scholars, Khulna FGD, 22 October 2022).

Changes in men's views were noteworthy, as reported by the scholars, “husbands now value their wives' decisions and listen to their opinions.” This was consistent with comments made by men throughout their FGDs, especially when discussing the changes in their wives after the training. Broader changes among the wives were observed by several men.

“My wife now has more confidence to make her own decisions. She took over some household decisions, especially those related to the kids. It helps reduce my burden so that I can focus more on other activities.” (a man FGD, Koyra, 15 October 2020).

“My wife has become more recognized in the family as well as in the community. Her situation has changed....the training has empowered her to make decisions at home.” (a man FGD, Dumuria, 16 October 2020).

To measure the extent to which the training has improved the health and wellbeing of the participating women and their children, the questionnaire asked men to rate the improvement on a scale from 1 to 10, with 1 indicating no improvement at all and 10 a very large improvement. The scores given by the respondents rendered a median of 8.5 and a mean of 7.7 (due to two low scores, 2 in Kaliganji and 3 in Tala), which indicates that the men highly valued the positive effects brought about by the training.

The above statements resonated with the community nutrition scholars' feelings over their journey in the project. They were asked to rate, on a scale between 1 and 10, the level to which their involvement as a community scholar had boosted their self-confidence and independence as decision-makers. The responses were overwhelmingly positive, with 75% (*n* = 20) of the scholars giving the highest score, while the rest assigned a 9 to the statement. Such high scores were anticipated from the beginning of the FGDs when the scholars described the transformation they underwent through the program.

“Before, I was not able to speak to anyone. Now I am comfortable to do that, and everyone listens to me. They respect me and take my advice.” (an FGD with community nutrition scholar, Satkhira, 21 October 2020).

“In the past, I could not gather the villagers or make them listen. I had to explain a lot to them. Slowly, I managed to get their attention and they started listening to me.” (an FGD with community nutrition scholars, Satkhira, 21 October 2020).

“I used to be inactive. Previously, nobody knew me. Now they respect me.”

“I am at ease to speak with anyone.”

“When I can give good advice to the mothers, I feel proud of myself.” (an FGD with community nutrition scholars, Khulna, 22 October 2020).

Finally, the following stories from in-depth interviews with community nutrition scholars are compelling examples of how the experiences in the project activities have improved their standing in the community. Pseudonyms are used to protect identities.

Champa, like many other married women in Tala, never left the home alone. In 2018, only 2 months before she joined the program, her husband passed away, leaving her to care for two children. Her participation as a community nutrition scholar, which involved interacting, speaking, and giving advice to various members of the village, has helped her build self-confidence as well as earn respect from others. Importantly, it gave her the strength to overcome the hard times she was facing.

“I have gained so much more self-confidence that I no longer fear speaking in public. Honestly, I would have been lost in the dark without this job. Now I can make money without having to ask anyone for support.” (In-depth interview, Tala, 23 March).

The sweet potato she grew from the provided OFSP vines enabled her to save money on food and make an additional income by selling the excess. Her focus now lies on her son and daughter; she wants to ensure they receive the education they need to build a secure future.

The changes in the husbands' attitudes toward their wives were also noticeable, as in the case of Sharmin.

“At first, my husband was not happy with me going out ‘door to door', afraid of what people would think of me. But eventually, after learning about my work, he was supportive and encouraged me to continue.” (In-depth interview, Koyra, 23 March 2021).

Working as a scholar, she earned a regular salary, which lifted the burden of household spending as well as her medication. Her husband allowed her to keep the salary and income from the sweet potato as her own, and she decided freely what to do with it. Occasionally, it was spent on jewelry and make-up for her daughter, who would not ask her father for such things. Moreover, Sharmin also managed to put aside a monthly amount of 1,000 Taka (12 USD) to a savings account at Grameen Bank. Her economic situation improved, but what is more meaningful is the change in her social position.

“Before the nutrition program, people never welcomed me in their houses. They did not spend time or listen to me. But now, since I gave them nutrition training, I've got respect from the community; they greet me wherever I see them.” (In-depth interview, Koyra, 23 March 2021).

The transformation was strongly felt by younger trainers. Afia, at the age of 26 years, was passionate when talking about the sense of pride and reward the program had cultivated in her. She has earned the community's respect, which extends beyond the life of the project.

“Being able to teach and mobilize mothers in the community, I feel proud of myself. Even now, all the women who participated in my training sessions still show me the same respect they had for me during the course. Whenever we see each other, they greet me and ask for my advice about childcare, nutrition, and other things. It brings me happiness. Thanks to the scholar job, my time management has improved, and the experience of working with different people in the community has given me the skills and confidence to interact with everyone, young and old, as well as influential people in our community.” (In-depth interview, Dumuria, 22 March 2021).

Krishna lost her father to a stroke 3 years ago. He used to be the sole breadwinner of the family. Currently, Krishna lives with her mother and two younger brothers. By working as a nutrition trainer, she was able to support her family.

“Now I'm contributing to my family. My opinions are considered in the[sic] household decision-making. It feels so good to be able to take some responsibility for my home.”

She has recently invested in a small chicken-raising plan, hoping to earn more income by selling eggs and chickens.

“Now I always look out for opportunities to invest and raise my earnings. Whenever my younger brothers need money, I give them some, it makes me happy. They would ask me because they know I am capable of helping out.”

“My relatives and family would like me to find a husband and settle down, but that's not [sic] I want now. I've got a degree which I worked very hard for, so I want to get established and do more. If I get married now, I might not be able to pursue further studies or work because the[sic] husband might be against it. Now my family respects my plan because I am working. They would have married me off if I was doing nothing and just sitting around. Since this community nutrition scholar program, I have been able to go out on my own comfortably. I am very happy.” (In-depth interview, Satkhira Sadar, 24 March 2021).

Krishna's standing in the community was also transformed:

“Before this, no one knew me. Now people know me, respect me, and consult me when their kids get sick, or ask me what food to feed the kids and such. As a community nutrition scholar, I can advise them, and this makes me so proud. Even with mothers much older than me, I am now capable of speaking with them and make them listen.”

The nutrition intervention had positive influences on both the community nutrition scholars and the trainees, in terms of self-confidence and position in the family as well as in the community.

## 4. Discussion

This qualitative assessment of the nutrition intervention explored (1) the processes and reasonings for changes in child feeding, food preparation, hygiene practices, and homestead gardening, (2) men's roles in facilitating women's behavioral changes, and (3) women's changes in their subjective notions of self-confidence, decision-making, and recognition.

The findings from the 14 FGDs and 6 in-depth interviews provide good evidence that the nutrition intervention with 5,000 mothers from two districts in southern Bangladesh resulted in changes in behavior by the women, their spouses, and other family members. Although there is a strong cultural pressure on participants in these kinds of assessments to minimize the negative and maximize the positive, the triangulation of three different perspectives on the same set of training events and implementation of the practices learned in the training provided additional assurance that the behavior changes and the gender transformation, in some cases, were authentic.

Many women who attended the training were able to ask their husbands to purchase nutritious food in local food markets or allocate home-produced eggs and milk for home consumption. As shown in Section 3.3, the women did not have such bargaining power before obtaining nutrition knowledge through the training. As a result of the training, women were listened to more by their family members, and they were better able to take independent actions that increased their self-confidence. Furthermore, trust in the training by the women's spouses and their mothers-in-law was equally important. Through this trust, women's voices were more valued than before, and they were able to make independent actions, changing food allocations based on recommendations from community nutrition scholars. Although it was not possible for this short-term evaluation to identify positive nutritional and health outcomes among the children, women increased decision-making power, self-confidence, and independence are positive indicators. The nutrition literature in Bangladesh suggests that women's intra-household bargaining power in relation to food purchases is significantly associated with children's weight (11, 13).

These findings also demonstrate that women devoted greater attention and time to child feeding, childcare, and food preparation. This may have a positive effect on the children's nutritional outcomes in the long term as the amount of time women spend with their children has a positive association with their children's nutritional status (44).

Alternatively, this assessment had a limited scope to explore women's time, labor burdens, and trade-offs. In the rural context, where cooking and water access lack labor-saving characteristics, childcare, water use, food preparation, and cooking are time-consuming for women. Women's time and labor burden in productive and reproductive activities have raised concerns for maternal health in South Asia (45, 46). Hence, men's greater involvement in child health and nutrition is essential in confronting these maternal health concerns. However, in development interventions and the Sustainable Development Goals, more attention appears to be given to women's equal involvement in economic activities than men's equal involvement in unpaid reproductive activities through nutrition and health interventions (47).

This study confirms that gender divisions of labor are not straightforward, and men, as well as other family members, are involved in parts of reproductive activities either as laborers to support the women or as decision-makers. Men exhibited positive perceptions and interests in nutrition interventions, and this result is encouraging to engage men in future nutrition interventions. Men's greater involvement in child health and nutrition can potentially facilitate more investment in this field, reducing women's time and labor burdens, and creating space for women to engage in economic activities. All are likely to contribute to better child nutrition outcomes.

Furthermore, men's perspectives on nutrition have implications for agricultural policy and interventions. In some study communities, men were interested in growing nutrition-rich crops, such as OFSP, beyond homestead gardens for home consumption. Currently, there is a shift in global food and agricultural policies from securing staple crop production to developing nutrition-sensitive cropping systems (48, 49). Given that men are usually farm managers in Bangladesh, men's involvement in nutrition interventions could help increase agricultural producer awareness of the importance of nutrition-sensitive food systems. This could increase support to plant more diverse and nutritious crops on farms. The topic of nutrition-sensitive food systems can be included in the module for men in future nutrition training.

Further in-depth research is required to better understand the complex intra-household power dynamics including gender and age-based relations, especially the role and influence of mothers-in-law on young mothers. To enrich nutrition scholarship, especially the pathways in which gender interacts with child nutrition and health, men's roles and influences, changing through the impacts of migration, urbanization, and other demographic and socio-economic drivers, warrant further investigation.

## 5. Conclusion

The underlying causes of micronutrient deficiencies are complex and include biological, social, environmental, and economic factors (6–9). Intra-household gender relations play a significant role in women's autonomy and decision-making power over household resource allocation and child feeding, hence child nutrition and health (26). However, much current nutrition research and interventions tend to focus on providing skills and knowledge to women and assessing their improved behavior and nutritional outcomes (16). Understanding nuanced intra-household gender relations with their spouses and other relatives is the first step in making nutrition interventions more effective, equitable, and inclusive (14, 15).

Our qualitative evaluation of the nutrition intervention in Southern Bangladesh highlights the way women's behavioral change following a nutrition education intervention is closely associated with their intra-household gender relations. Men play significant roles in facilitating their wives' behavioral change, such as purchasing nutritious food from local markets which women are culturally discouraged from visiting, allocating their own-produced milk and eggs to home consumption, providing labor to home garden preparation and production, and defending their spouses from mothers-in-law who are often resistant to change. Men's indirect contribution to child health and nutrition should be more clearly recognized in nutrition scholarship as it helps improve the effectiveness of nutrition interventions and can even encourage greater gender equality in the divisions of labor within and outside the household. Narratives from women community nutrition scholars show that this peer-based, women-to-women training approach has a great potential to strengthen women's self-confidence and recognition in the household and the community. Our study thus presented nuanced processes in which women's improved nutritional skills and knowledge bring synergies between improving child nutrition and women's position. This is a major contribution of our study to nutrition scholarship, especially, in relation to social development goal 5: Achieve gender equality and empower all women and girls.

## Data availability statement

The raw data supporting the conclusions of this article will be made available by the authors, without undue reservation.

## Author contributions

GP, NK, FI, and ER contributed to the study conception and design. FI and ER performed data collection and contributed to writing the sections of manuscripts. GP conducted a first analysis. NK conducted a literature review. GP and NK wrote the first draft of the manuscript. All authors contributed to manuscript revisions and read and approved the submitted version.
